# A Highly Selective and Sensitive Fluorescent Sensor Based on Molecularly Imprinted Polymer-Functionalized Mn-Doped ZnS Quantum Dots for Detection of Roxarsone in Feeds

**DOI:** 10.3390/nano12172997

**Published:** 2022-08-30

**Authors:** Fei Li, Jie Gao, Haocheng Wu, Yijun Li, Xiwen He, Langxing Chen

**Affiliations:** 1 College of Chemical Engineering and Materials Science, Shandong Normal University, Jinan 250014, China; 2 Tianjin Key Laboratory of Biosensing and Molecular Recognition, College of Chemistry, Nankai University, Tianjin 300071, China; 3 National Demonstration Center for Experimental Chemistry Education, Nankai University, Tianjin 300071, China

**Keywords:** roxarsone, molecularly imprinted polymers, Mn-doped ZnS quantum dots, feeds

## Abstract

Roxarsone (ROX) as an organoarsenic feed additive has been widely used in livestock breeding and poultry industry, but ROX can degrade into highly toxic inorganic arsenic species in natural environments to threaten to the environment and human health. Therefore, there is a considerable interest in developing convenient, selective and sensitive methods for the detection of ROX in livestock breeding and poultry industry. In this work, a fluorescent molecularly imprinted polymer (MIPs) probe based on amino-modified Mn-ZnS quantum dots (QDs) has been developed by sol–gel polymerization for specific recognition of ROX. The synthesized MIPs-coated Mn-ZnS QDs (MIPs@Mn-ZnS QDs) have highly selective recognition sites to ROX because there are multi-interactions among the template ROX, functional monomer phenyltrimethoxysilane and the amino-functionalized QDs such as the π–π conjugating effect, hydrogen bonds. Under the optimal conditions, an obvious fluorescence quenching was observed when ROX was added to the solution, and the quenching mechanism could be explained as the photo-induced electron transfer. The MIPs@Mn-ZnS QDs sensor exhibited sensitive response to ROX in the linear range from 3.75 × 10^−8^ M to 6.25 × 10^−7^ M (R^2^ = 0.9985) and the limit of detection down to 4.34 nM. Moreover, the fluorescence probe has been applied to the quantitative detection of ROX in feed samples, and the recovery was in the range of 91.9% to 108.0%. The work demonstrated that the prepared MIPs@Mn-ZnS QDs probe has a good potential for rapid and sensitive determination of ROX in complicated samples.

## 1. Introduction

As the most economical organoarsenic feed additives, roxarsone (ROX) has been widely used in the livestock breeding and poultry industry because it can promote animal growth [1,2,3], anti-coccidial intestinal parasites [4,5] and cure dysentery and sedimentary pigments [6,7]. In 1944, ROX was allowed for use in chicken feed by the United States Food and Drug Administration (FDA) and was officially approved by the Ministry of Agriculture of China as a growth-promoting agent for pigs and chickens in 1996. However, as a representative of water-soluble organic arsenic, ROX could be degraded into inorganic arsenic with high toxicity when it enters the environment with animal droppings [8,9]. In order to guarantee the quality and safety of the animal production database and maintain public health and ecological safety, ROX was prohibited as a feed additive by the European Union in 1999, the USA in 2011 and China in 2018, sequentially, but it was still broadly used in other developing countries. Therefore, there is great interest to develop a convenient, selective and sensitive method for the precise detection of ROX in feed samples.

At present, some methods have been reported for ROX detection, such as electroanalytical method [10,11,12], gas chromatography (GC) [13], high-performance liquid chromatography (HPLC) [14], mass spectrometry [15] and hyphenated instruments (GC-MS and HPLC-MS) [16,17,18,19,20,21]. These approaches show satisfactory sensitivity and also share some shortcomings, such as miscellaneous sample pretreatment, being time-consuming, large amounts of sample demand and sophisticated equipment, which limited their use in routine animal products monitoring. Fluorescent spectroscopy is a potential alternative method due to its low cost, short analysis time and simple equipment. In recent years, QDs have been extensively used in the field of biochemistry because of their unique photochemical properties (quantum size effect, good photostability, large stokes shift, good biocompatibility and narrow and symmetric emission spectrum) [22,23]. However, most of the QDs contain heavy metal elements such as cadmium and mercury which would render the environment hazardous [24,25] and limit their applications. Mn-ZnS QDs have attracted many scholars due to their low toxicity and resistance to photobleaching, which made them more suitable for biochemical sensing and cell imaging [26].

In recent years, a highly effective fluorescent probe based on core-shell-structured QD molecularly imprinted polymers (MIPs) have been reported for small molecules and macromolecular detection [27,28,29,30]. Due to their specific recognition, facile preparation, cost-effectiveness, physical inertness and excellent reusability, MIPs have aroused widespread attention in the fields of solid phase extraction, chromatographic separation, biocatalysis and pharmaceutical analysis [31,32]. Sol–gel polymerization is a process that hydrolyzes and condenses alcohol-based precursors to make polysiloxane networks connected by siloxane bonds between silanol groups. Compared with organic polymerization for MIPs, sol–gel systems have good water-solubility [33,34], mild temperature processability, are easy to deposit on different substrates and provide precise control of the polymerization process [35].

Recently, Zhang et al. [36] prepared ROX-imprinted polymers and chose arsanilic acid as the dummy template and applied solid phase extraction. Fan et al. [37] synthesized MIPs for selective recognition of ROX with UV–Vis spectra. In this work, we fabricated a fluorescent probe based on MIPs-coated amino-modified Mn-ZnS QDs for ROX detection with high selectivity and sensitivity by sol–gel polymerization. The optical and adsorption performance of MIPs@Mn-ZnS QDs were explored. The condition on the detection of ROX was also investigated. Furthermore, the fluorescent probe based on MIPs@Mn-ZnS QDs was successfully applied for the detection of ROX in feed samples.

## 2. Experimental Section

### 2.1. Materials and Methods

Manganese sulfate tetrahydrate (MnSO_4_∙H_2_O), ammonium hydroxide (NH_3_∙H_2_O), sodium sulfide nonahydrate (Na_2_S∙9H_2_O) and zinc sulfate heptahydrate (ZnSO_4_∙7H_2_O) were purchased from Tianjin Chemical Reagent Company (Tianjin, China). Nitroxynil (NIT), (3-Aminopropy) triethoxysilane (APTES) and 2-methoxy-5-nitrophenol (NSD) were received from Aladdin Reagent Company (Shanghai, China). 4-Aminophenylarsonic acid (ASA) was purchased from Energy Chemical (Shanghai, China). Phenyltrimethoxysilane (PTMOS) and tetraethylorthosilicate (TEOS) were provided by Alfa Aesar (Tianjin, China). 3-Mercaptopropy trimethoxysilane (MPTS) was obtained from Tokyo Chemical Industry (Tokyo, Japan). 3-Acetamido-4-hydroxy-phenylarsonic Acid (ACA) and 4-hydroxy-3-nitrophenylarsonic Acid (ROX) were provided by Heowns (Tianjin, China) and TCI (Shanghai, China). All other chemical regents were of analytical grade and no further purification was performed. Ultra-pure water used in this work was prepared with a Aquapro Ultrapure Water System AFZ-6000-U (Chongqing, China).

### 2.2. Instruments and Characterizations

The Fourier transform infrared spectra (FT-IR) of nanoparticles were measured with the AVATAR 360 FT-IR spectrophotometer (Nicolet, Waltham, MA, USA) in KBr with the range of 4000–400 cm^−1^. The distribution and size of the nanoparticles were measured by transmission electron microscope (TEM) with a JEM-2800 (JEOL Ltd., Tokyo, Japan). The crystalline phase of the QDs were measured by the X-ray diffractometer (XRD) on a Rigaku D/Max-2500 diffractometer (Rigaku Corporation, Tokyo, Japan) with a range of 3° < 2θ < 80° at a rate of 4° min^−1^, which used the monochromatized X-ray beam with nickel-filtered Cu K_α_ radiation. The surface potential of the QDs was tested by a Zeta potential Analyzer ZETAPALS/BI-200SM (Brookhaven Instruments Corporation, Holtsville, NY, USA). The chemical composition of the synthesized QDs was measured by X-ray photoelectron spectroscopy (XPS) on Kratos AXIS Ultra DLD system (Kratos Analytical Ltd., Manchester, UK). All fluorescence spectra were performed on F-4600 fluorescence spectrophotometer from Hitachi (Hitachi Ltd., Tokyo, Japan).

### 2.3. Preparation of MPTS Functionalized Mn-ZnS QDs (MPTS@Mn-ZnS QDs)

The synthesis route of MPTS@Mn-ZnS QDs was adapted from previous work of our research group [38]. MnSO_4_∙H_2_O (0.845 g, 0.5 mmol) and ZnSO_4_∙7H_2_O (1.8 g, 6.25 mmol) were dissolved in ultra-pure water of 40 mL with 100 mL boiling flask-3-neck, stirring 10 min under the protection of argon and 10 mL aqueous solution containing 6.25 mmol Na_2_S∙9H_2_O was added, stirring for 30 min, followed by the addition of 10 mL anhydrous ethanol containing 100 μL MPTS and then stirring for 20 h. The mixture was separated by centrifugation at 10,000 rpm, washed three times with ultra-pure water and anhydrous ethanol, respectively, then dried in a 50 °C vacuum oven for 12 h.

### 2.4. Preparation of Amino Modified Mn-ZnS QDs (APTES@Mn-ZnS QDs)

The amino-modified Mn-ZnS QDs were synthesized referring to the previous literature [39]. A total of 0.2 g Mn-ZnS@MPTS QDs was uniformly dispersed in 25 mL round-bottomed flask with 10 mL anhydrous ethanol solution, stirred under argon for 10 min, and then 350 μL TEOS and 200 μL APTES were added. After stirring for 10 min, 2.0 mL ultra-pure water containing 400 μL ammonium hydroxide was added and stirred for 18 h. Finally, the obtained mixture was centrifugated at 10,000 rpm and washed with anhydrous ethanol thrice and then dried in a 50 °C vacuum oven for 12 h.

### 2.5. Preparation of MIPs@Mn-ZnS QDs and NIPs

A total of 10 mg of ROX and 36.8 μL PTMOS as functional monomer were dispersed into 20 mL of absolute ethanol/water (*v*/*v*, 1:1) solution in a 25 mL round-bottomed flask, then 20 mg APTES@Mn-ZnS QDs was dissolved homogeneously in 10 mL ultra-pure water and kept stirring for 30 min to obtain prepolymer at ambient temperature. Then, 169 μL TEOS was added as cross linker. After stirring for 10 min, 150 μL NH_3_∙H_2_O as catalyst was added and kept stirred for 24 h. The resultants were centrifugated at 10,000 rpm and washed with anhydrous ethanol. The template molecules were extracted by the mixed solvent of triethylamine/methanol (*v*/*v*, 10:1) and repeated several times until no ROX in the supernatant was detected by UV–Vis spectrophotometer. Finally, MIPs@Mn-ZnS QDs were dried in a 50 °C vacuum oven for 12 h. The non-imprinted polymers (NIPs@Mn-ZnS QDs) were also prepared by the same procedure with no ROX added.

### 2.6. Preparation of Feed Samples

Typically, accurately weighted feed of 2 g was transferred into a 50 mL centrifugal tube, 20 mL methanol–water (*v*/*v* = 1:1) was added, the mixture was homogenized for 2 min, then put in an ultrasonic water bath for 40 min. Subsequently, the mixture was centrifuged 10 min at 12,000 rpm, the supernatant was transferred into a 50 mL centrifugal tube. The sediment in the centrifuge was extracted once. The two supernatants were combined together, filtered through 0.45 μm filter, and evaporated at 60 °C to dryness.

### 2.7. Fluorescence Measurement

All the fluorescence spectra were measured as follows: the width of the excitation and emission slit is 10 nm, the voltage of the photomultiplier tube is 700 V, the excitation wavelength is 310 nm, the emission wavelength is in the range of 400–800 nm and the scanning speed is 2400 nm·min^−1^. The MIPs@Mn-ZnS and NIPs@Mn-ZnS QDs were uniformly dispersed in ultra-pure water by ultrasonic to obtain 1 mg·mL^−1^ fresh-made stock solution. A series of ROX standard solutions with various concentration were added to the 100 μL dispersion solution of MIPs@Mn-ZnS or NIPs@Mn-ZnS QDs and the volume fixed to 1 mL to investigate the quenching ability of the prepared nanomaterials towards ROX.

## 3. Results and Discussion

### 3.1. Preparation of MIPs Coated Mn-ZnS QDs (MIPs@Mn-ZnS QDs)

**Figure 1:**
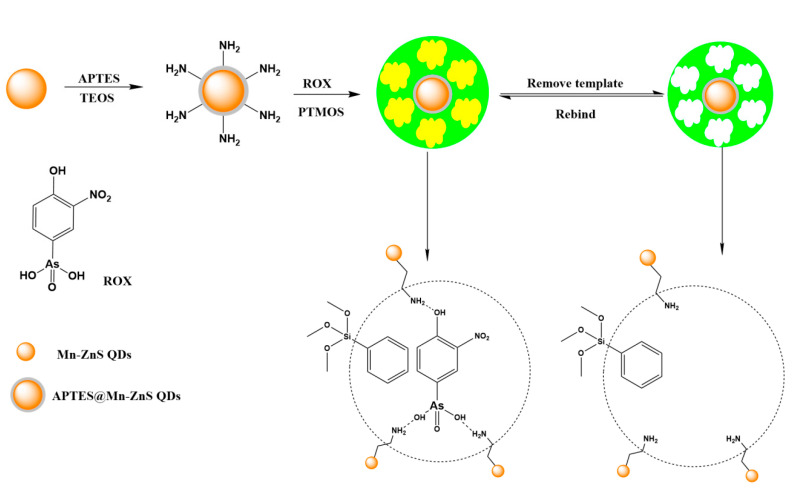
Schematic illustration of the preparation procedure of MIPs@Mn-ZnS QDs for ROX.

The synthesis of Mn-ZnS QDs functionalized by MIPs for ROX was schematized in Figure 1. Firstly, the MPTS-functionalized Mn-ZnS QDs was synthesized according to the literature [38]. Silica coating had been demonstrated as an ideal shell to protect the QDs. The chemical inertia and optical transparence of MPTS could render the thin and nonopaque protective layer on the surface of Mn-ZnS QDs. Secondly, amino groups were modified on the surface of MPTS-coated Mn-ZnS QDs. The amino-coated Mn-ZnS QDs not only acted as solid support but also as a functional monomer introducing template molecules into silica arrays because the amino groups could enhance the interaction between ROX and QDs through hydrogen bond interactions. The core-shell MIPs@Mn-ZnS QDs were prepared by the hydrolytic condensation of PTMOS and TEOS in the sol–gel polymerization process with ammonia as catalyst. After ROX was removed, the imprinted cavities matched ROX in size, and orientation and functional groups were formed.

### 3.2. Optimization of Synthesis Condition for MIPs@Mn-ZnS QDs

**Figure 2:**
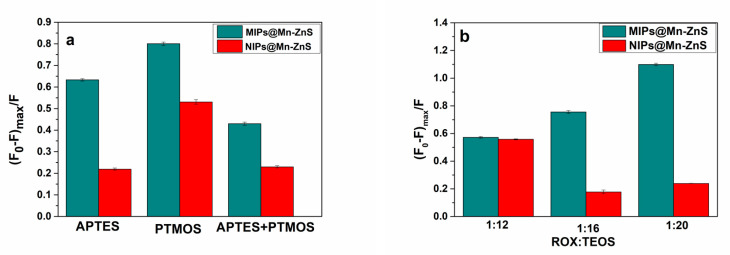
The effect of the different type of functional monomer (**a**), and the molar ratio of ROX to cross-linker (TEOS) (**b**) on the fluorescence intensity of MIPs@Mn-ZnS QDs towards ROX.

The binding capacity of MIPs@Mn-ZnS QDs to ROX was mainly dependent on the amount of recognition sites in the MIP layer. In order to find the best proportion of polymerization for MIPs@Mn-ZnS QDs, the type of functional monomer, the ratio of template to cross-linking agent were investigated. On the basis of the structure characteristics of ROX, the functional monomers which could better match with target molecules were explored. In this study, APTES-modified Mn-ZnS QDs could offer binding sites to react with hydroxyl and arsonic acid groups of the template. PTMOS, APTES and the mixture of APTES and PTMOS (1:1, n/n) were chosen as functional monomers, respectively. The binding ability of the corresponding polymers was investigated by the quenching efficiency of MIPs@Mn-ZnS QDs. As shown in Figure 2a, when PTMOS was chosen as the functional monomer, the fluorescence quenching efficiency was the best compared with APTES or the mixture of APTES and PTMOS. This might result from the multi-interactions existing in the matrix of APTES@Mn-ZnS QDs, target molecule and monomer. The amine groups modified on APTES@Mn-ZnS QDs could provide hydrogen bonds with oxygen atoms and bind with arsenic acid through the acid-based interaction [40], while PTMOS provided phenyl to react with the phenyl residue of ROX through π–π conjugation. Theoretically, bifunctional monomers can provide more interactions, but they also cause considerable steric hindrance, which is not conducive to the formation of imprinted sites. Thus, in the following experiments, PTMOS was chosen as the monomer.

The amount of cross-linking agent played a significant part in the imprinting effect. When the content of cross-linking agent was too little in the reaction solution, the MIPs could not maintain a stable three-dimensional structure due to the low degree of crosslinking. However, while the usage of cross-linking agent was too much, the imprint layer coated on the surface of the QDs was thick, resulting in the low fluorescence intensity of MIPs and little specific recognition sites. Figure 2b shows the influence of the molar ratio of target molecule to cross-linking agent on the fluorescence intensity of MIPs@Mn-ZnS QDs. It was found that 1:20 was the best molar ratio for target molecule to cross-linker.

### 3.3. Charaterization of Fluorescent MIPs@Mn-ZnS QDs

**Figure 3:**
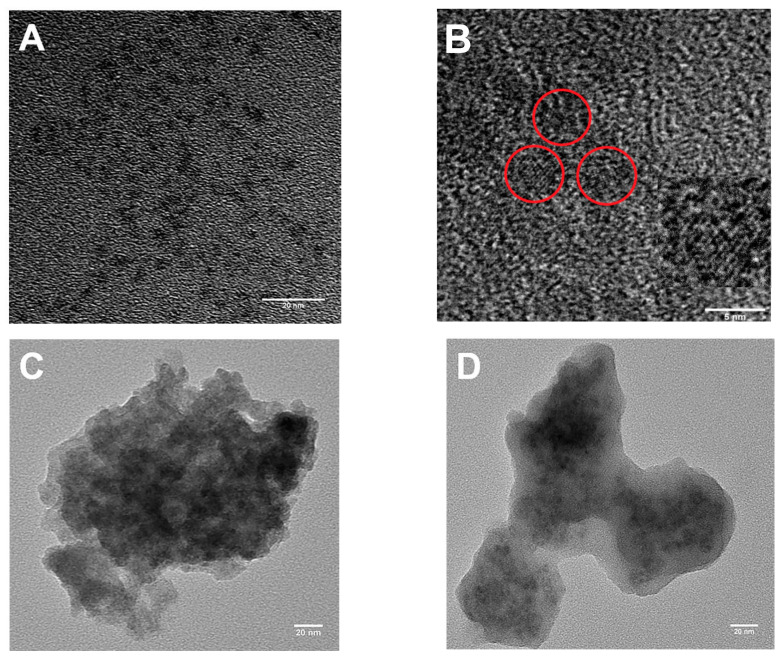
TEM images of the prepared APTES@Mn-ZnS QDs (**A**) and HRTEM images of APTES@Mn-ZnS QDs (**B**), MIPs@Mn-ZnS QDs (**C**) and NIPs@Mn-ZnS QDs (**D**).

TEM images of the APTES@Mn-ZnS, MIPs@Mn-ZnS and NIPs@Mn-ZnS were shown in Figure 3. It could be seen that the APTES@Mn-ZnS QDs (Figure 3A) were uniformly distributed in a sphere with the diameter of 3.5 nm, approximately. Figure 3B shows the high-resolution transmission electron microscopy (HRTEM) of APTES@Mn-ZnS; it could be observed that the spacing of the crystal lattice fringe was 0.232 nm (red circle), which could be attributed to the (111) plane of Mn-ZnS QDs. The TEM image in Figure 3C,D shows the MIPs@Mn-ZnS and NIPs@Mn-ZnS core@shell particles, which were formed by the hydrolysis and condensation reaction of PTMOS and TEOS on the surface of the APTES@Mn-ZnS through sol–gel polymerization. It indicated that the Mn-ZnS QDs were successfully embedded into the irregular amorphous silicon. The results show that the repeated sol–gel polymerization could increase the thickness of the silica film.

**Figure 4:**
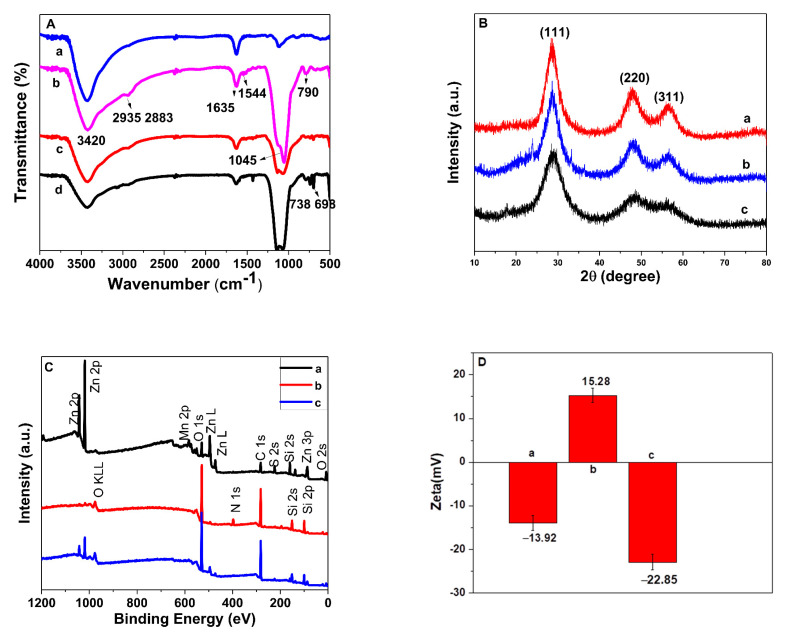
(**A**)FT-IR of MPTS@Mn-ZnS QDs (a), APTES@Mn-ZnS QDs (b), MIPs@Mn-ZnS QDs (c) and NIPs@Mn-ZnS QDs (d). (**B**) The XRD patterns of MIPs@Mn-ZnS QDs (a), APTES@Mn-ZnS QDs (b) and MPTS@Mn-ZnS QDs (c). (**C**) The XPS of MPTS@Mn-ZnS QDs (a), APTES@Mn-ZnS QDs (b) and MIPs@Mn-ZnSQDs (c). (**D**) The Zeta potentials of MPTS@Mn-ZnS (a), APTES@Mn-ZnS (b) and MIPs@Mn-ZnS QDs (c).

The FT-IR spectra were used to explore step-by-step surface chemical modification. The MPTS@Mn-ZnS QDs are shown in Figure 4A(a). The intensive and wide peak around 1045 cm^−1^ was attributed to the Si–O–Si asymmetric stretching vibration; the bands at 790 cm^−1^ could also be distinguished, which was attributed to the bending vibrations of Si–O. After modification with APTES, the absorption peaks at 2935 cm^−1^ and 2883 cm^−1^ could be assigned to the asymmetric and symmetric stretching of the C-H in the alkyl chain (Figure 4A(b)). The N-H stretching vibrations at 1544 cm^−1^ further revealed the successful modification of amino groups to the surface of the Mn-ZnS QDs. The above bands showed the successful synthesis of APTES@Mn-ZnS QDs. Compared with APTES@Mn-ZnS, the characteristic absorption peak at 738 cm^−1^ and 698 cm^−1^ of MIPs@Mn-ZnS and NIPs@Mn-ZnS represented the out-of-plane bending vibration of phenyl groups, indicating that the PTMOS had been successfully formed on the surface of APTES@Mn-ZnS QDs. In addition, curve 4A(c) and 4A(d) had almost the same characteristic peaks, indicating ROX had been completely removed.

The X-ray diffraction patterns of MPTS@Mn-ZnS (a), APTES@Mn-ZnS (b) and MIPs@Mn-ZnS (c) QDs are shown in Figure 4B. The crystal structure of three samples exhibited face-centered cubic sphalerite, the peaks assigned to the (1 1 1), (2 2 0) and (3 1 1), which matched with the standard card JCPDS NO.05-0566. The results confirm the Mn-ZnS QDs were successfully synthesized, and the similar shape of peaks indicated that the surface modification did not change the crystal structure of QDs. The diffraction peak intensity of MPTS@Mn-ZnS, APTES@Mn-ZnS and MIP@Mn-ZnS QDs decreased gradually, which indicated that the silicon layer on the surface of QDs was increased along with more sol–gel condensations.

The X-ray photoelectron spectroscopy was performed to explore the surface chemical composition of MPTS@Mn-ZnS, APTES@Mn-ZnS and MIPs@Mn-ZnS QDs. The XPS spectra (curve a to curve c in Figure 4C) exhibited the intense signals of Si 2p at 164.3 eV, S 2p at 99.7 eV, C 1s at 285 eV, O 1s at 532 eV, Mn 2p at 638 eV and Zn 2p at 1019 eV on the surface of three kinds of synthesized Mn-ZnS QDs. The enhanced signal of C 1s and O 1s, and the new appeared peak of N 1s at 397 eV in the XPS spectra of APTES@Mn-ZnS (Figure 4C(b)), MIPs@Mn-ZnS QDs (Figure 4C(c)), showed that the APTES and MIP layers were successfully modified onto the surface of the MPTS@Mn-ZnS QDs.

The zeta potentials of MPTS@Mn-ZnS, APTES@Mn-ZnS and MIPs@Mn-ZnS QDs are shown in Figure 4D. The zeta potential of MPTS@Mn-ZnS QDs was −13.92 ± 1.73 mV. After modified with APTES, the zeta potential increased to 15.28 ± 1.59 mV, which demonstrated the successful introduction of –NH_2_ groups to the surface of MPTS@Mn-ZnS. The zeta potential of MIP@Mn-ZnS QDs was changed into −22.85 ± 1.84 mV; this might be ascribed to the presence of a large density of hydroxyl groups on the surface of MIPs@Mn-ZnS QDs formed by hydrolysis of silane in the imprinted process. The result was consistent with the TEM image of MIPs@Mn-ZnS QDs.

### 3.4. The Optimization of the Fluorescent Measurement of MIPs@Mn-ZnS QDs to ROX

**Figure 5:**
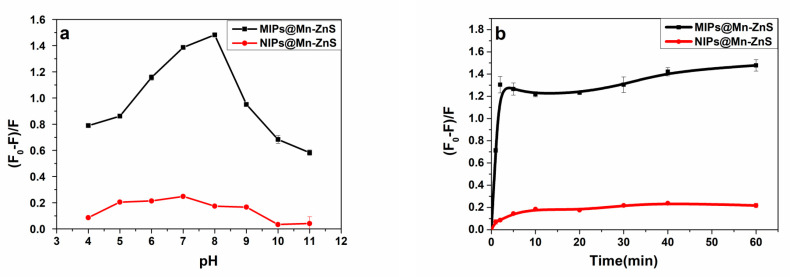
(**a**) The pH-dependent fluorescent quenching ratios changes of MIPs@Mn-ZnS QDs and NIPs@Mn-ZnS QDs in the presence of 2.5 µM ROX. (**b**) The effect of incubation time between the analyte (ROX) and MIPs@Mn-ZnS QDs on the fluorescence intensity of MIPs@Mn-ZnS QDs.

The fluorescent intensity of MIPs@Mn-ZnS QDs towards ROX molecules in aqueous media depends on a variety of interactions. For example, hydrogen binding, ionic interactions and hydrophobic effects. The influence of pH on the quenching ability of MIP@Mn-ZnS QDs towards ROX was investigated. The quenching ratio of the MIP@Mn-ZnS QDs in the pH range from 4 to 11 was investigated. As shown in Figure 5a. The fluorescence quenching ratio of the MIP@Mn-ZnS QDs to ROX was increased with pH from 4 to 8 and then decreased from 8 to 11. The phenomenon could be explained as follows: There were many amino groups in the MIP layer, and so the low response of the MIPs@Mn-ZnS QDs to ROX at low pH values was probably related to the protonation of amino groups. When pH was high, the hydroxyl anion would create a surface defect to damage the structure of the cavities because the imprinting layer could be ionized in alkaline environment [41], which would weaken the interaction between the MIPs and ROX. There were no special binding sites on the NIP@Mn-ZnS QDs, thus the influence of pH on the NIP@Mn-ZnS was little. On the whole, the pH of 7 was chosen for the subsequent experiment.

The appropriate incubation time between analyte (ROX) and MIPs@Mn-ZnS QDs also influenced the fluorescence intensity of MIPs@Mn-ZnS QDs. The fluorescence quenching ratios at different incubation time are shown in Figure 5b. After ROX was added into the solution of MIPs@Mn-ZnS QDs, the fluorescence quenching ratio of MIPs@Mn-ZnS QDs was sharply increased in the first two minutes and then remained roughly stable in the ensuing 58 min. However, it took ten minutes to keep stable for the fluorescence quenching ratio of NIPs@Mn-ZnS QDs. The incubation time of 10 min was chosen as the detection time of ROX in the follow-up experiments.

### 3.5. The Fluorescent Measurement of the MIPs@Mn-ZnS QDs towards ROX

**Figure 6:**
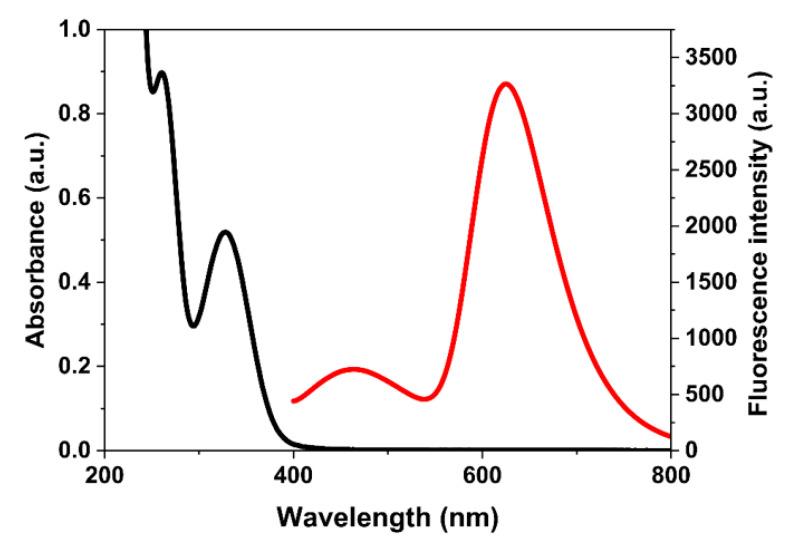
The UV–Vis absorption of ROX (black line) and fluorescence emission of APTES@Mn-ZnS QDs (red line).

There was no overlap between the UV–Vis absorption spectrum of ROX (black line) and the fluorescence emission spectrum of MIPs@Mn-ZnS QDs (red line) in the Figure 6, which did not meet exactly the condition of FRET. Therefore, the mechanism of Förster resonance energy transfer (FRET) between ROX and MIPs@Mn-ZnS QDs could be excluded. The groups of nitro and the arsenic acid in ROX were all electron-deficient units, however, the amino groups of the MIPs@Mn-ZnS QDs were electron-rich units and electrons could transfer from the MIPs@Mn-ZnS QDs to ROX through their strong binding to the template molecule, causing the quenching of the MIPs@Mn-ZnS QDs. The electrons were excited from the valence band to the conduction band and transited to the initial condition to generate the emissions. Therefore, the mechanism might be attributed to photo-induced electron transfer (PET). 

### 3.6. Specificity Evaluation of the MIPs@Mn-ZnS QDs

**Figure 7:**
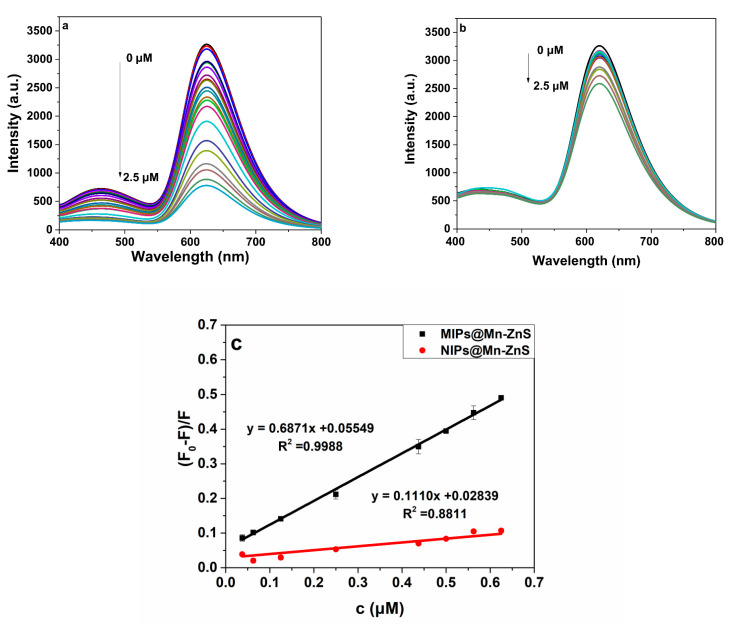
The fluorescence spectra of MIPs@Mn-ZnS QDs (**a**) and NIPs@Mn-ZnS QDs (**b**) in the presence of different concentrations of ROX. The linear calibration graph of MIPs@Mn-ZnS QDs and NIPs@Mn-ZnS QDs (**c**) in the presence of ROX.

The specific recognition ability of MIPs@Mn-ZnS and NIPs@Mn-ZnS towards ROX was investigated. The fluorescent response of MIPs@Mn-ZnS and NIPs@Mn-ZnS towards ROX at different concentrations is shown in Figure 7a,b. The quenching phenomenon in this system could be interpreted by the Stern–Volmer equation:

F_0_/F = K_SV_ C_q_ + 1 (1)

where F_0_ is the fluorescence intensity without ROX, F is the fluorescence intensity of different concentrations of ROX, C_q_ is the concentration of the quenching agent ROX in MIPs@Mn-ZnS or NIPs@Mn-ZnS QDs solution and K_SV_ is the Stern–Volmer quenching constant of the ROX. When the concentration of the ROX was in the range from 3.75 × 10^−8^ M to 6.25 × 10^−7^ M, the linear equation was (F_0_ – F)/F = 0.6871C_q_ + 0.05549 (R^2^ = 0.9988) and (F_0_ – F)/F = 0.1110C_q_ + 0.02839 (R^2^ = 0.8811) for MIPs@Mn-ZnS and NIPs@Mn-ZnS, respectively. The limit of detection (LOD) was calculated by the formula of LOD = 3σ/*k*, and the limit of quantification (LOQ) was calculated by the equation LOQ = 10σ/*k,* where *k* is the slope of the linear calibration, and σ is the standard deviation of blank signal. According to the above equations, the LOD and LOQ were 4.34 nM and 14.4 nM for MIPs@Mn-ZnS QDs, respectively. In comparison with other reported quantitative methods for ROX [10,14,18,19,20,21,42] (Table 1), the LOD in this work is lower than that of the electroanalytical and UPLC method, almost equivalent to HPLC-ICP-MS, but higher than that of HPLC-AFS.

Additionally, the imprinting factor (IF) was usually used to estimate the specific property of MIPs@Mn-ZnS and NIPs@Mn-ZnS QDs towards ROX. The IF was defined as the proportion of K_SV_ of MIPs to NIPs (K_SV(MIPs)_/K_SV(NIPs)_), and K_SV(MIPs)_ and K_SV(NIPs)_ were the linear slope of MIPs and NIPs, respectively (Figure 7c) [43]. Under the optimal condition, the IF value was estimated to 6.19, which suggested that the MIPs@Mn-ZnS QDs had excellent specificity for ROX detection.

**Figure 8:**
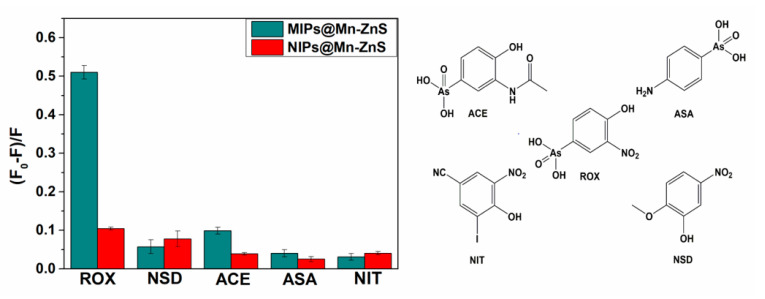
(F_0_ − F)/F of MIPs@Mn-ZnS QDs and NIPs@Mn-ZnS QDs in the presence of ROX, NSD, ACE, ASA and NIT (the concentration of analyte is 0.5 µM for ROX and its analogues).

The selectivity of the MIPs@Mn-ZnS QDs for ROX was tested by competitive experiments. The structure analogues ASA, NSD, ACA and NIT, which were structurally related with the target molecule ROX, were chosen for evaluating the fluorescence quenching of MIPs@Mn-ZnS and NIPs@Mn-ZnS QDs in the same concentration (0.5 μM). The fluorescence quenching ratios of MIPs@Mn-ZnS and NIPs@Mn-ZnS QDs in the presence of ROX, ASA, NSD, ACA and NIT are displayed in Figure 8. The fluorescence quenching ratios of MIPs@Mn-ZnS QDs to ROX were much higher than those of the analogues, but the NIPs@Mn-ZnS did not exhibit significant difference to ROX and the analogues. The reason might be due to the tailor-made recognition sites which matched with the template in size, shape and space. It could be supposed that the electron-accepting character of the nitro group in the roxarsone affects the acidity of the compound. The higher acidity of roxarsone (because of the presence of the nitro group in meta position) with respect to acetamido group in ACE could be responsible for higher specificity due to electrostatic interactions with the amine group in the polymer network. This was also why 4-amino-derivative ASA, which was characterized by significantly lower acidity when compared with roxarsone, was characterized by low specificity [44,45].

### 3.7. Detection of ROX in the Feed Samples

The feasibility of the proposed method in the real samples was investigated. As shown in Table 2, there was no fluorescence response without spiked ROX in the feed sample. The recoveries of ROX for three spiked feed samples were in the range of 91.9–108.0%, with a relative standard deviation (RSD) ranging from 2.2% to 3.9%. The results display that MIPs@Mn-ZnS QDs could successfully identify and analyze ROX in the feed samples.

## 4. Conclusions

In this work, a new strategy for specific identification of ROX based on fluorescent MIPs@Mn-ZnS QDs was proposed. The MIPs@Mn-ZnS QDs as eco-friendly nanoprobes have the merits of the unique optical properties of semiconductor QDs and the high selectivity of MIPs. The MIPs@Mn-ZnS QDs exhibited specific selectivity to ROX over other structure analogues and can sensitively detect down to 4.34 nM, and there is a linear relationship between 3.75 × 10^−8^ M and 6.25 × 10^−7^ M for ROX. The results exhibit that the prepared MIPs@Mn-ZnS QDs could detect ROX rapidly, selectively and sensitively. The performance in the detection of ROX in feed samples with good recoveries and reproducibility demonstrated the method had the potential to detect the ROX rapidly in complicated samples.

## Figures and Tables

**Table 1:** Comparison of the proposed method with the other reported methods for determination of ROX *.

Detection Method	Detection Range (nM)	LOD (nM)	LOQ (nM)	Refs.
Electroanalytical	2000–100,000	528	1760	[12]
UPLC-UV	760–19,000	380	760	[16]
HPLC-ICP-MS	0–760	5.78	-	[20]
HPLC-ICP-MS	3.8–380	5.49	18	[21]
HPLC-ICP-MS	3.8–6.8	4.56	12.2	[22]
HPLC-UV/AFS	19–1520	342	1140	[23]
LC-HG-AFS	760–15,209	760	-	[42]
Fluorescence	37.5–625	4.33	14.4	This work

* The data provided in Table 1 have been consistent and have the same units in mass for a proper comparison.

**Table 2:** Recovery (mean ± s; n = 3) for the determination of ROX in the feed samples.

Samples	ROX Spiked (10^−7^ M)	ROX Found (10^−7^ M)	Recovery (%)
Feed 1	0.00	-	-
Feed 2	0.80	0.86	108.0 ± 3.9
Feed 3	4.00	4.10	101.4 ± 2.2
Feed 4	6.00	5.50	91.9 ± 2.5

## Data Availability

The data presented in this study are available on request from the corresponding author.
